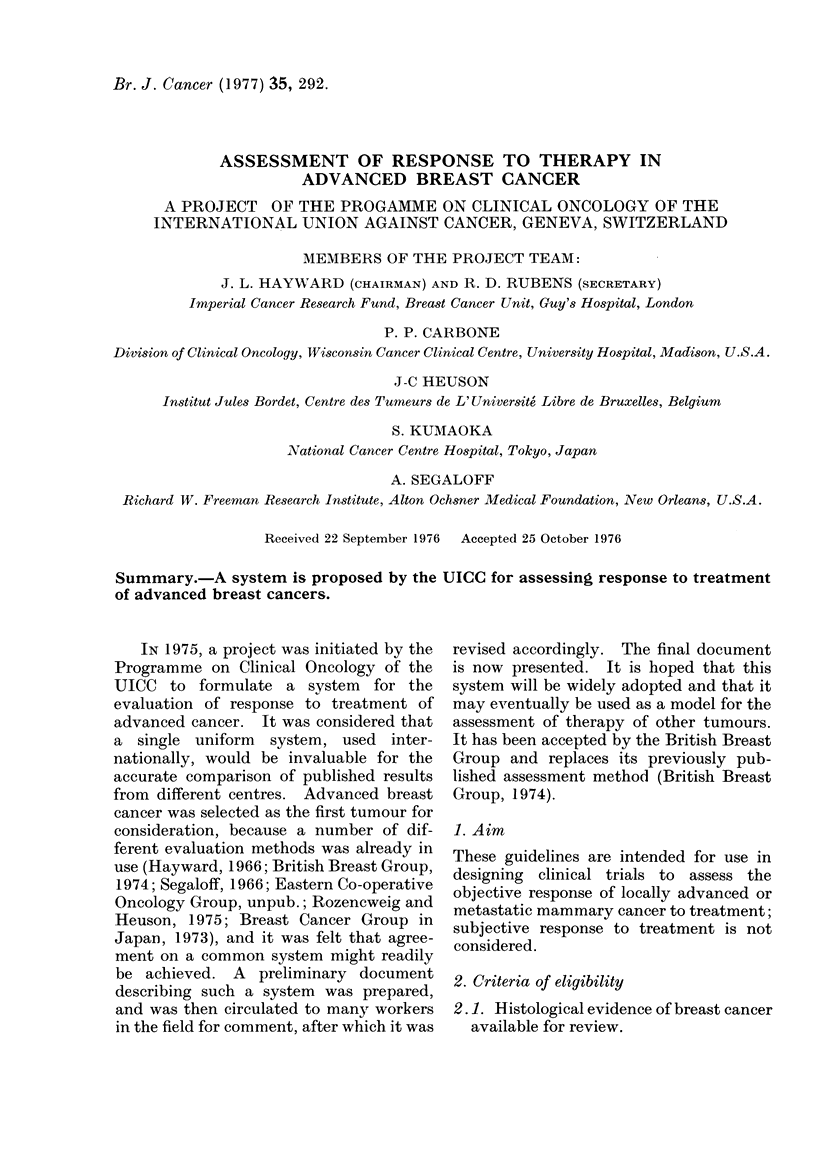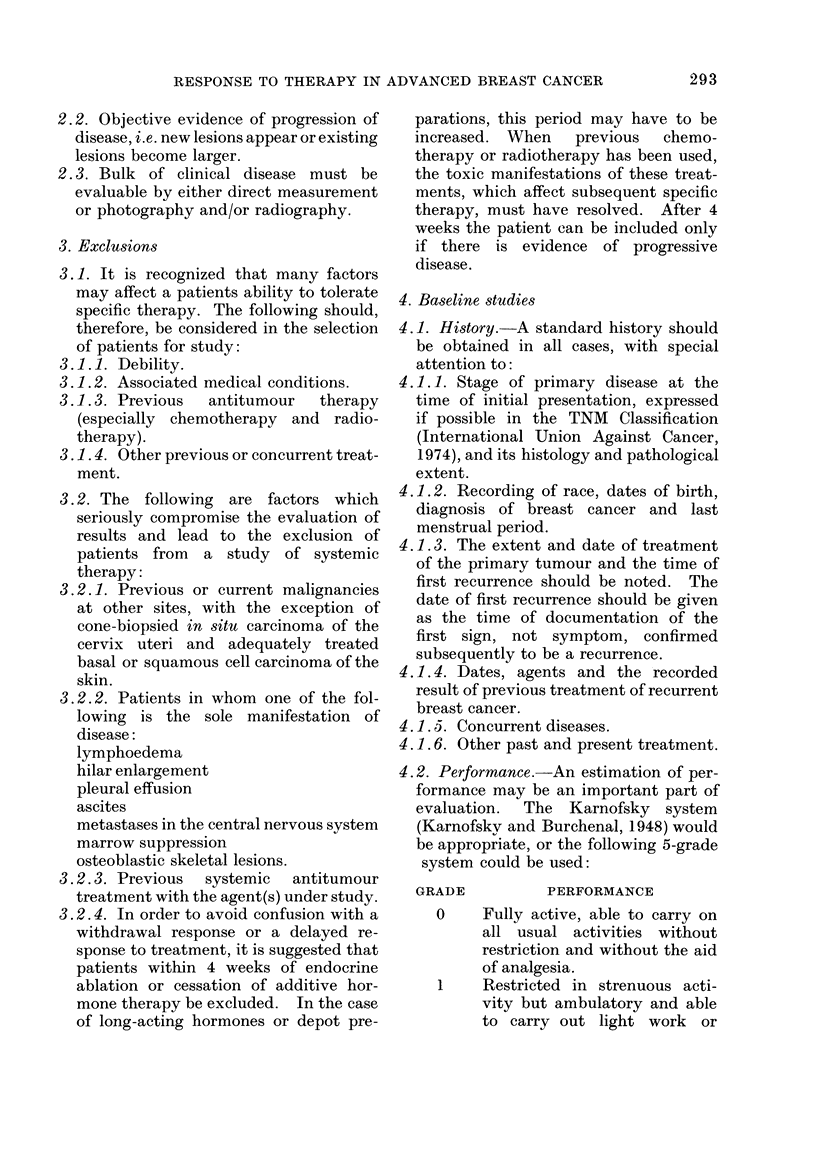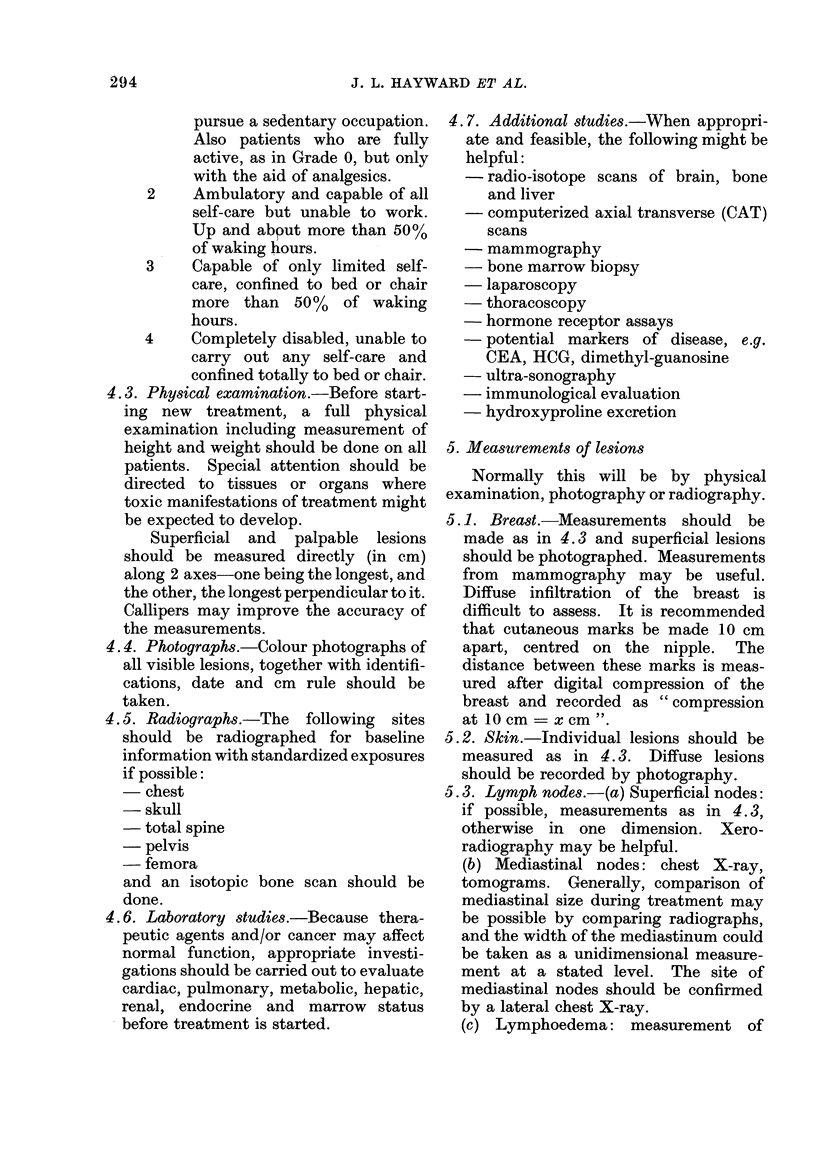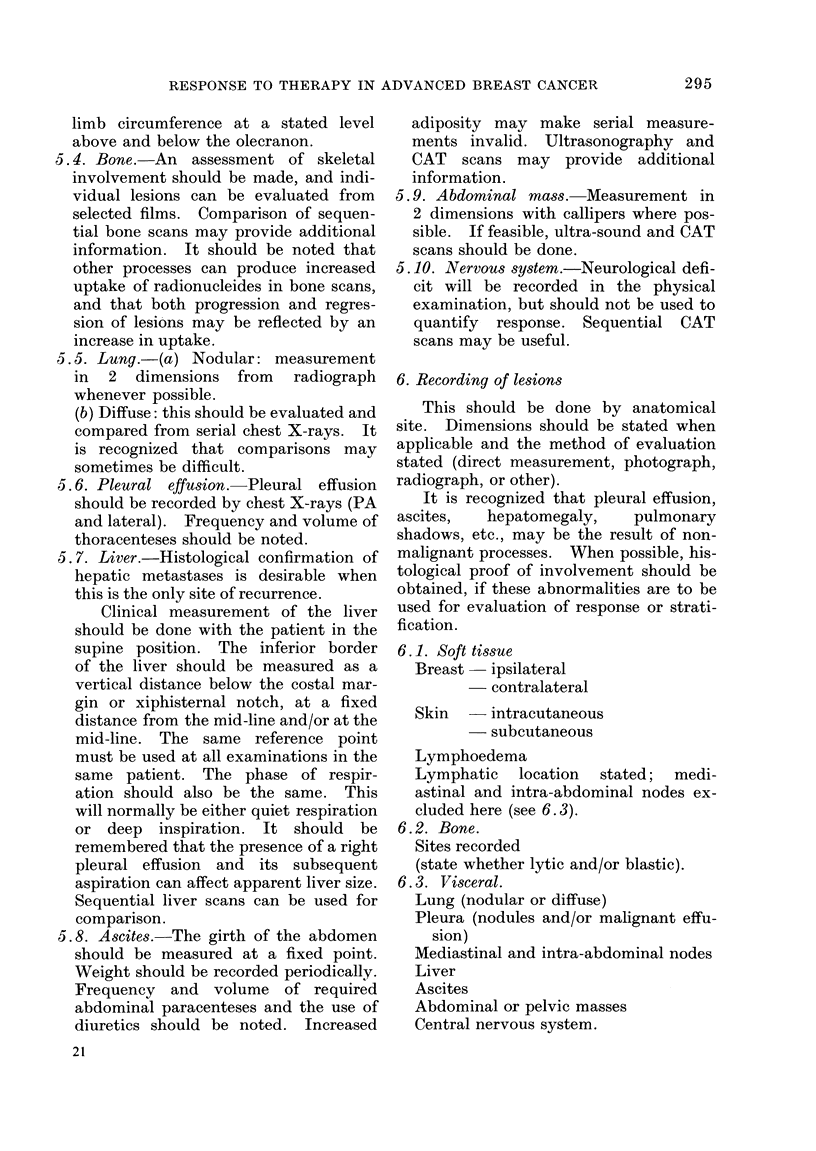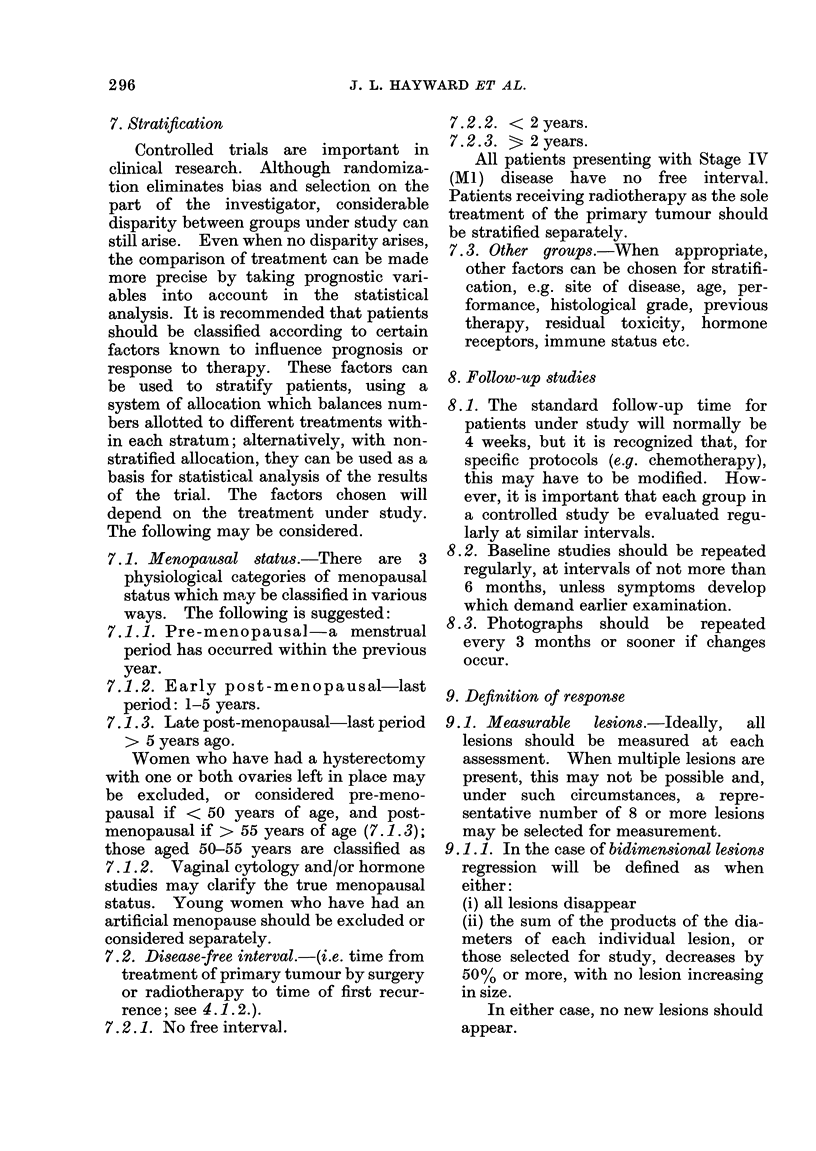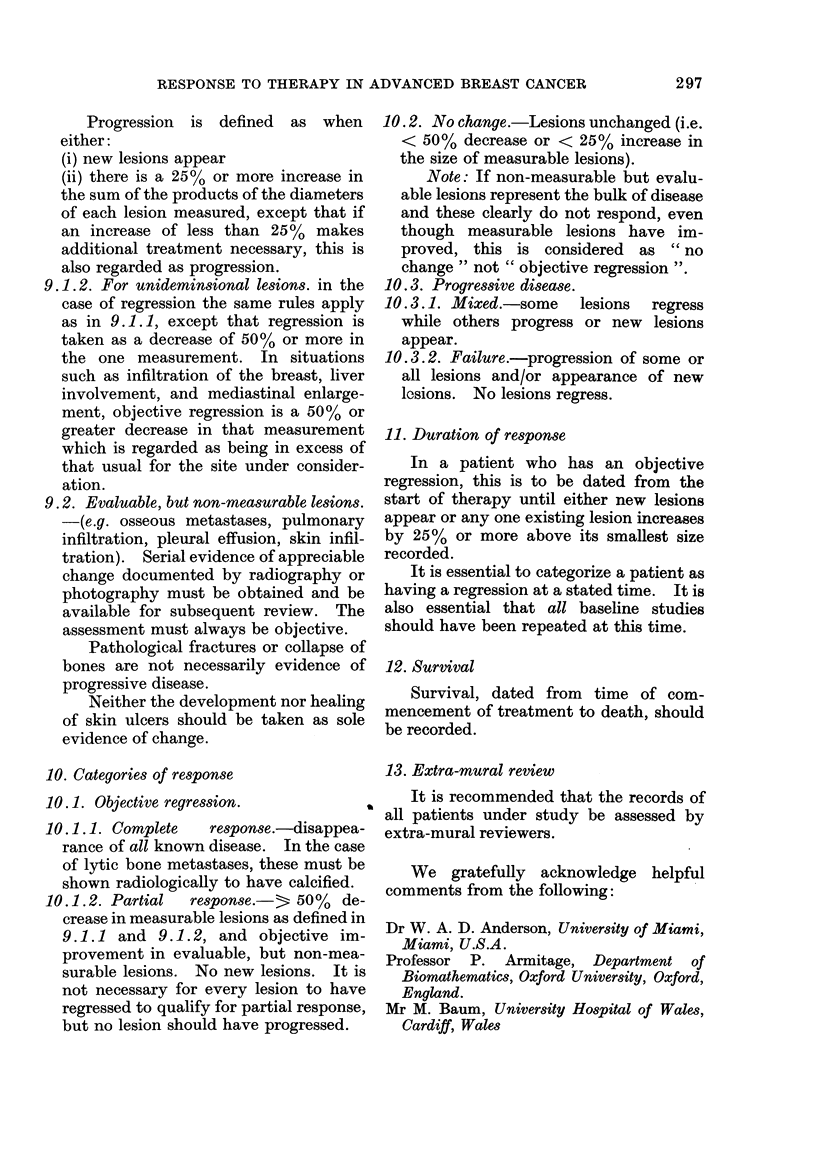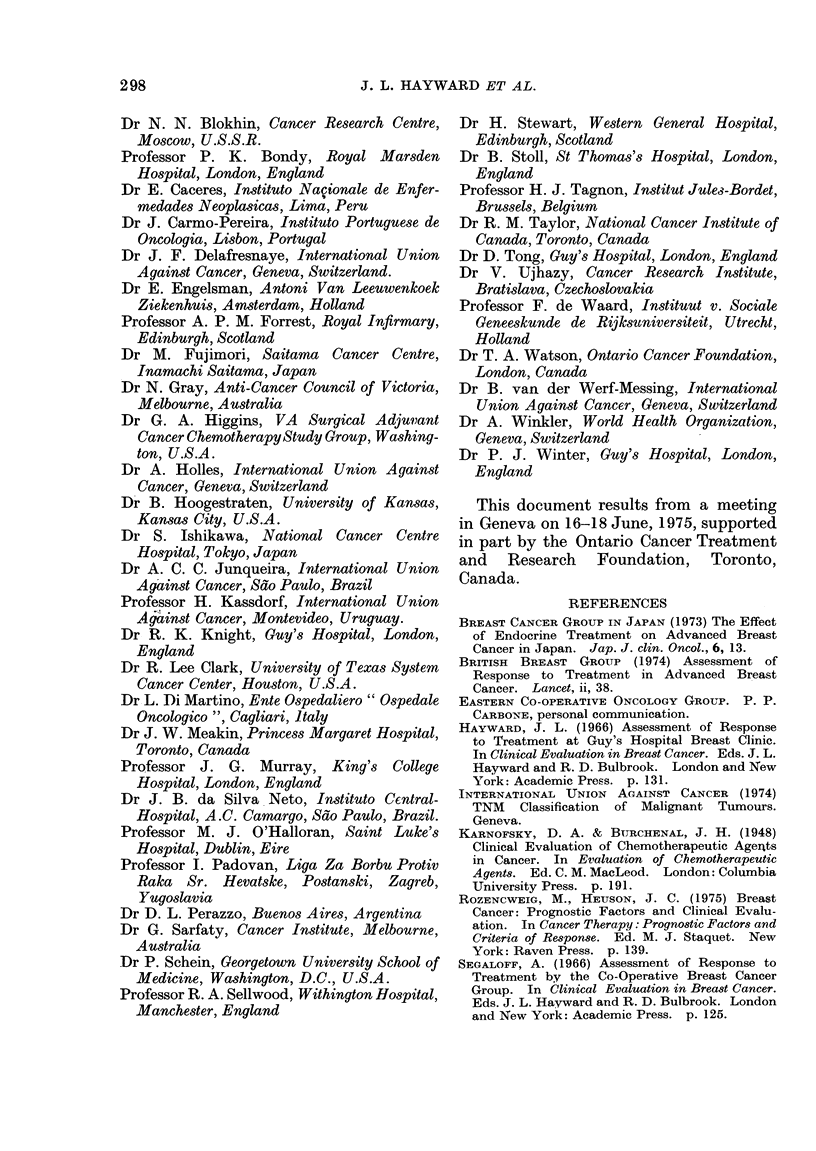# Assessment of response to therapy in advanced breast cancer.

**DOI:** 10.1038/bjc.1977.42

**Published:** 1977-03

**Authors:** J. L. Hayward, P. P. Carbone, J. C. Heusen, S. Kumaoka, A. Segaloff, R. D. Rubens

## Abstract

A system is proposed by the UICC for assessing response to treatment of advanced breast cancers.


*Br. J. Cancer* (1977) **35**, 292.

### ASSESSMENT OF RESPONSE TO THERAPY IN ADVANCED BREAST CANCER

### A PROJECT OF THE PROGAMME ON CLINICAL ONCOLOGY OF THE INTERNATIONAL UNION AGAINST CANCER, GENEVA, SWITZERLAND

MEMBERS OF THE PROJECT TEAM:

J. L. HAYWARD (CHAIRMAN) AND R. D. RUBENS (SECRETARY)

*Imperial Cancer Research Fund, Breast Cancer Unit, Guy's Hospital, London*

P. P. CARBONE

*Division of Clinical Oncology, Wisconsin Cancer Clinical Centre, University Hospital, Madison, U.S.A.*

J-C HEUSON

*Institut Jules Bordet, Centre des Tumeurs de L'Université Libre de Bruxelles, Belgium*

S. KUMAOKA

*National Cancer Centre Hospital, Tokyo, Japan*

A. SEGALOFF

*Richard W. Freeman Research Institute, Alton Ochsner Medical Foundation, New Orleans, U.S.A.*

Received 22 September 1976 Accepted 25 October 1976

**Summary.—A system is proposed by the UICC for assessing response to treatment of advanced breast cancers.**

IN 1975, a project was initiated by the Programme on Clinical Oncology of the UICC to formulate a system for the evaluation of response to treatment of advanced cancer. It was considered that a single uniform system, used internationally, would be invaluable for the accurate comparison of published results from different centres. Advanced breast cancer was selected as the first tumour for consideration, because a number of different evaluation methods was already in use (Hayward, 1966; British Breast Group, 1974; Segaloff, 1966; Eastern Co-operative Oncology Group, unpub.; Rozencweig and Heuson, 1975; Breast Cancer Group in Japan, 1973), and it was felt that agreement on a common system might readily be achieved. A preliminary document describing such a system was prepared, and was then circulated to many workers in the field for comment, after which it was revised accordingly. The final document is now presented. It is hoped that this system will be widely adopted and that it may eventually be used as a model for the assessment of therapy of other tumours. It has been accepted by the British Breast Group and replaces its previously published assessment method (British Breast Group, 1974).

### *1. Aim*

These guidelines are intended for use in designing clinical trials to assess the objective response of locally advanced or metastatic mammary cancer to treatment; subjective response to treatment is not considered.

### *2. Criteria of eligibility*

*2.1.* Histological evidence of breast cancer available for review.

*2.2.* Objective evidence of progression of disease, *i.e.* new lesions appear or existing lesions become larger.
*2.3.* Bulk of clinical disease must be evaluable by either direct measurement or photography and/or radiography.

*3. Exclusions*

*3.1.* It is recognized that many factors may affect a patients ability to tolerate specific therapy. The following should, therefore, be considered in the selection of patients for study:
*3.1.1.* Debility.
*3.1.2.* Associated medical conditions.
*3.1.3.* Previous antitumour therapy (especially chemotherapy and radiotherapy).
*3.1.4.* Other previous or concurrent treatment.

*3.2.* The following are factors which seriously compromise the evaluation of results and lead to the exclusion of patients from a study of systemic therapy:
*3.2.1.* Previous or current malignancies at other sites, with the exception of cone-biopsied *in situ* carcinoma of the cervix uteri and adequately treated basal or squamous cell carcinoma of the skin.
*3.2.2.* Patients in whom one of the following is the sole manifestation of disease:
lymphoedema
hilar enlargement
pleural effusion
ascites
metastases in the central nervous system
marrow suppression
osteoblastic skeletal lesions.
*3.2.3.* Previous systemic antitumour treatment with the agent(s) under study.
*3.2.4.* In order to avoid confusion with a withdrawal response or a delayed response to treatment, it is suggested that patients within 4 weeks of endocrine ablation or cessation of additive hormone therapy be excluded. In the case of long-acting hormones or depot preparations, this period may have to be increased. When previous chemotherapy or radiotherapy has been used, the toxic manifestations of these treatments, which affect subsequent specific therapy, must have resolved. After 4 weeks the patient can be included only if there is evidence of progressive disease.

*4. Baseline studies*

*4.1. History.*—A standard history should be obtained in all cases, with special attention to:
*4.1.1.* Stage of primary disease at the time of initial presentation, expressed if possible in the TNM Classification (International Union Against Cancer, 1974), and its histology and pathological extent.
*4.1.2.* Recording of race, dates of birth, diagnosis of breast cancer and last menstrual period.
*4.1.3.* The extent and date of treatment of the primary tumour and the time of first recurrence should be noted. The date of first recurrence should be given as the time of documentation of the first sign, not symptom, confirmed subsequently to be a recurrence.
*4.1.4.* Dates, agents and the recorded result of previous treatment of recurrent breast cancer.
*4.1.5.* Concurrent diseases.
*4.1.6.* Other past and present treatment.

*4.2. Performance.*—An estimation of performance may be an important part of evaluation. The Karnofsky system (Karnofsky and Burchenal, 1948) would be appropriate, or the following 5-grade system could be used:

| GRADE | PERFORMANCE |
|---|---|
| 0 | Fully active, able to carry on all usual activities without restriction and without the aid of analgesia. |
| 1 | Restricted in strenuous activity but ambulatory and able to carry out light work or |

pursue a sedentary occupation. Also patients who are fully active, as in Grade 0, but only with the aid of analgesics.

2 Ambulatory and capable of all self-care but unable to work. Up and about more than 50% of waking hours.

3 Capable of only limited self-care, confined to bed or chair more than 50% of waking hours.

4 Completely disabled, unable to carry out any self-care and confined totally to bed or chair.

*4.3. Physical examination.*—Before starting new treatment, a full physical examination including measurement of height and weight should be done on all patients. Special attention should be directed to tissues or organs where toxic manifestations of treatment might be expected to develop.

Superficial and palpable lesions should be measured directly (in cm) along 2 axes—one being the longest, and the other, the longest perpendicular to it. Callipers may improve the accuracy of the measurements.

*4.4. Photographs.*—Colour photographs of all visible lesions, together with identifications, date and cm rule should be taken.

*4.5. Radiographs.*—The following sites should be radiographed for baseline information with standardized exposures if possible:

— chest
— skull
— total spine
— pelvis
— femora and an isotopic bone scan should be done.

*4.6. Laboratory studies.*—Because therapeutic agents and/or cancer may affect normal function, appropriate investigations should be carried out to evaluate cardiac, pulmonary, metabolic, hepatic, renal, endocrine and marrow status before treatment is started.

*4.7. Additional studies.*—When appropriate and feasible, the following might be helpful:

— radio-isotope scans of brain, bone and liver
— computerized axial transverse (CAT) scans
— mammography
— bone marrow biopsy
— laparoscopy
— thoracoscopy
— hormone receptor assays
— potential markers of disease, *e.g.* CEA, HCG, dimethyl-guanosine
— ultra-sonography
— immunological evaluation
— hydroxyproline excretion

*5. Measurements of lesions*

Normally this will be by physical examination, photography or radiography.

*5.1. Breast.*—Measurements should be made as in *4.3* and superficial lesions should be photographed. Measurements from mammography may be useful. Diffuse infiltration of the breast is difficult to assess. It is recommended that cutaneous marks be made 10 cm apart, centred on the nipple. The distance between these marks is measured after digital compression of the breast and recorded as "compression at 10 cm = $x$ cm".

*5.2. Skin.*—Individual lesions should be measured as in *4.3.* Diffuse lesions should be recorded by photography.

*5.3. Lymph nodes.*—(*a*) Superficial nodes: if possible, measurements as in *4.3*, otherwise in one dimension. Xeroradiography may be helpful.

(*b*) Mediastinal nodes: chest X-ray, tomograms. Generally, comparison of mediastinal size during treatment may be possible by comparing radiographs, and the width of the mediastinum could be taken as a unidimensional measurement at a stated level. The site of mediastinal nodes should be confirmed by a lateral chest X-ray.

(*c*) Lymphoedema: measurement of limb circumference at a stated level above and below the olecranon.

5.4. *Bone.*—An assessment of skeletal involvement should be made, and individual lesions can be evaluated from selected films. Comparison of sequential bone scans may provide additional information. It should be noted that other processes can produce increased uptake of radionucleides in bone scans, and that both progression and regression of lesions may be reflected by an increase in uptake.

5.5. *Lung.*—(*a*) Nodular: measurement in 2 dimensions from radiograph whenever possible.
(*b*) Diffuse: this should be evaluated and compared from serial chest X-rays. It is recognized that comparisons may sometimes be difficult.

5.6. *Pleural effusion.*—Pleural effusion should be recorded by chest X-rays (PA and lateral). Frequency and volume of thoracenteses should be noted.

5.7. *Liver.*—Histological confirmation of hepatic metastases is desirable when this is the only site of recurrence.

Clinical measurement of the liver should be done with the patient in the supine position. The inferior border of the liver should be measured as a vertical distance below the costal margin or xiphisternal notch, at a fixed distance from the mid-line and/or at the mid-line. The same reference point must be used at all examinations in the same patient. The phase of respiration should also be the same. This will normally be either quiet respiration or deep inspiration. It should be remembered that the presence of a right pleural effusion and its subsequent aspiration can affect apparent liver size. Sequential liver scans can be used for comparison.

5.8. *Ascites.*—The girth of the abdomen should be measured at a fixed point. Weight should be recorded periodically. Frequency and volume of required abdominal paracenteses and the use of diuretics should be noted. Increased adiposity may make serial measurements invalid. Ultrasonography and CAT scans may provide additional information.

5.9. *Abdominal mass.*—Measurement in 2 dimensions with callipers where possible. If feasible, ultra-sound and CAT scans should be done.

5.10. *Nervous system.*—Neurological deficit will be recorded in the physical examination, but should not be used to quantify response. Sequential CAT scans may be useful.

6. *Recording of lesions*

This should be done by anatomical site. Dimensions should be stated when applicable and the method of evaluation stated (direct measurement, photograph, radiograph, or other).

It is recognized that pleural effusion, ascites, hepatomegaly, pulmonary shadows, etc., may be the result of non-malignant processes. When possible, histological proof of involvement should be obtained, if these abnormalities are to be used for evaluation of response or stratification.

6.1. *Soft tissue*

Breast — ipsilateral
— contralateral

Skin — intracutaneous
— subcutaneous

Lymphoedema
Lymphatic location stated; mediastinal and intra-abdominal nodes excluded here (see *6.3*).

6.2. *Bone.*

Sites recorded
(state whether lytic and/or blastic).

6.3. *Visceral.*

Lung (nodular or diffuse)
Pleura (nodules and/or malignant effusion)
Mediastinal and intra-abdominal nodes
Liver
Ascites
Abdominal or pelvic masses
Central nervous system.

*7. Stratification*

Controlled trials are important in clinical research. Although randomization eliminates bias and selection on the part of the investigator, considerable disparity between groups under study can still arise. Even when no disparity arises, the comparison of treatment can be made more precise by taking prognostic variables into account in the statistical analysis. It is recommended that patients should be classified according to certain factors known to influence prognosis or response to therapy. These factors can be used to stratify patients, using a system of allocation which balances numbers allotted to different treatments within each stratum; alternatively, with non-stratified allocation, they can be used as a basis for statistical analysis of the results of the trial. The factors chosen will depend on the treatment under study. The following may be considered.

*7.1. Menopausal status.*—There are 3 physiological categories of menopausal status which may be classified in various ways. The following is suggested:

*7.1.1.* Pre-menopausal—a menstrual period has occurred within the previous year.

*7.1.2.* Early post-menopausal—last period: 1–5 years.

*7.1.3.* Late post-menopausal—last period $> 5$ years ago.

Women who have had a hysterectomy with one or both ovaries left in place may be excluded, or considered pre-menopausal if $< 50$ years of age, and post-menopausal if $> 55$ years of age (*7.1.3*); those aged 50–55 years are classified as *7.1.2.* Vaginal cytology and/or hormone studies may clarify the true menopausal status. Young women who have had an artificial menopause should be excluded or considered separately.

*7.2. Disease-free interval.*—(*i.e.* time from treatment of primary tumour by surgery or radiotherapy to time of first recurrence; see *4.1.*2.).

*7.2.1.* No free interval.

*7.2.2.* $< 2$ years.

*7.2.3.* $\geqslant 2$ years.

All patients presenting with Stage IV (M1) disease have no free interval. Patients receiving radiotherapy as the sole treatment of the primary tumour should be stratified separately.

*7.3. Other groups.*—When appropriate, other factors can be chosen for stratification, e.g. site of disease, age, performance, histological grade, previous therapy, residual toxicity, hormone receptors, immune status etc.

*8. Follow-up studies*

*8.1.* The standard follow-up time for patients under study will normally be 4 weeks, but it is recognized that, for specific protocols (*e.g.* chemotherapy), this may have to be modified. However, it is important that each group in a controlled study be evaluated regularly at similar intervals.

*8.2.* Baseline studies should be repeated regularly, at intervals of not more than 6 months, unless symptoms develop which demand earlier examination.

*8.3.* Photographs should be repeated every 3 months or sooner if changes occur.

*9. Definition of response*

*9.1. Measurable lesions.*—Ideally, all lesions should be measured at each assessment. When multiple lesions are present, this may not be possible and, under such circumstances, a representative number of 8 or more lesions may be selected for measurement.

*9.1.1.* In the case of *bidimensional lesions* regression will be defined as when either:

(i) all lesions disappear

(ii) the sum of the products of the diameters of each individual lesion, or those selected for study, decreases by 50% or more, with no lesion increasing in size.

In either case, no new lesions should appear.

Progression is defined as when either:
(i) new lesions appear
(ii) there is a 25% or more increase in the sum of the products of the diameters of each lesion measured, except that if an increase of less than 25% makes additional treatment necessary, this is also regarded as progression.

*9.1.2. For unideminsional lesions.* in the case of regression the same rules apply as in *9.1.1*, except that regression is taken as a decrease of 50% or more in the one measurement. In situations such as infiltration of the breast, liver involvement, and mediastinal enlargement, objective regression is a 50% or greater decrease in that measurement which is regarded as being in excess of that usual for the site under consideration.

*9.2. Evaluable, but non-measurable lesions.* —(*e.g.* osseous metastases, pulmonary infiltration, pleural effusion, skin infiltration). Serial evidence of appreciable change documented by radiography or photography must be obtained and be available for subsequent review. The assessment must always be objective.

Pathological fractures or collapse of bones are not necessarily evidence of progressive disease.

Neither the development nor healing of skin ulcers should be taken as sole evidence of change.

*10. Categories of response*

*10.1. Objective regression.*

*10.1.1. Complete response.*—disappearance of *all* known disease. In the case of lytic bone metastases, these must be shown radiologically to have calcified.

*10.1.2. Partial response.*—$\geqslant$ 50% decrease in measurable lesions as defined in *9.1.1* and *9.1.2*, and objective improvement in evaluable, but non-measurable lesions. No new lesions. It is not necessary for every lesion to have regressed to qualify for partial response, but no lesion should have progressed.

*10.2. No change.*—Lesions unchanged (i.e. $<$ 50% decrease or $<$ 25% increase in the size of measurable lesions).

*Note:* If non-measurable but evaluable lesions represent the bulk of disease and these clearly do not respond, even though measurable lesions have improved, this is considered as " no change " not " objective regression ".

*10.3. Progressive disease.*

*10.3.1. Mixed.*—some lesions regress while others progress or new lesions appear.

*10.3.2. Failure.*—progression of some or all lesions and/or appearance of new lesions. No lesions regress.

*11. Duration of response*

In a patient who has an objective regression, this is to be dated from the start of therapy until either new lesions appear or any one existing lesion increases by 25% or more above its smallest size recorded.

It is essential to categorize a patient as having a regression at a stated time. It is also essential that *all* baseline studies should have been repeated at this time.

*12. Survival*

Survival, dated from time of commencement of treatment to death, should be recorded.

*13. Extra-mural review*

It is recommended that the records of all patients under study be assessed by extra-mural reviewers.

We gratefully acknowledge helpful comments from the following:

Dr W. A. D. Anderson, *University of Miami, Miami, U.S.A.*
Professor P. Armitage, *Department of Biomathematics, Oxford University, Oxford, England.*
Mr M. Baum, *University Hospital of Wales, Cardiff, Wales*

Dr N. N. Blokhin, *Cancer Research Centre, Moscow, U.S.S.R.*
Professor P. K. Bondy, *Royal Marsden Hospital, London, England*
Dr E. Caceres, *Instituto Naçionale de Enfermedades Neoplasicas, Lima, Peru*
Dr J. Carmo-Pereira, *Instituto Portuguese de Oncologia, Lisbon, Portugal*
Dr J. F. Delafresnaye, *International Union Against Cancer, Geneva, Switzerland.*
Dr E. Engelsman, *Antoni Van Leeuwenkoek Ziekenhuis, Amsterdam, Holland*
Professor A. P. M. Forrest, *Royal Infirmary, Edinburgh, Scotland*
Dr M. Fujimori, *Saitama Cancer Centre, Inamachi Saitama, Japan*
Dr N. Gray, *Anti-Cancer Council of Victoria, Melbourne, Australia*
Dr G. A. Higgins, *VA Surgical Adjuvant Cancer Chemotherapy Study Group, Washington, U.S.A.*
Dr A. Holles, *International Union Against Cancer, Geneva, Switzerland*
Dr B. Hoogestraten, *University of Kansas, Kansas City, U.S.A.*
Dr S. Ishikawa, *National Cancer Centre Hospital, Tokyo, Japan*
Dr A. C. C. Junqueira, *International Union Against Cancer, São Paulo, Brazil*
Professor H. Kassdorf, *International Union Against Cancer, Montevideo, Uruguay.*
Dr R. K. Knight, *Guy's Hospital, London, England*
Dr R. Lee Clark, *University of Texas System Cancer Center, Houston, U.S.A.*
Dr L. Di Martino, *Ente Ospedaliero " Ospedale Oncologico ", Cagliari, Italy*
Dr J. W. Meakin, *Princess Margaret Hospital, Toronto, Canada*
Professor J. G. Murray, *King's College Hospital, London, England*
Dr J. B. da Silva Neto, *Instituto Central-Hospital, A.C. Camargo, São Paulo, Brazil.*
Professor M. J. O'Halloran, *Saint Luke's Hospital, Dublin, Eire*
Professor I. Padovan, *Liga Za Borbu Protiv Raka Sr. Hevatske, Postanski, Zagreb, Yugoslavia*
Dr D. L. Perazzo, *Buenos Aires, Argentina*
Dr G. Sarfaty, *Cancer Institute, Melbourne, Australia*
Dr P. Schein, *Georgetown University School of Medicine, Washington, D.C., U.S.A.*
Professor R. A. Sellwood, *Withington Hospital, Manchester, England*
Dr H. Stewart, *Western General Hospital, Edinburgh, Scotland*
Dr B. Stoll, *St Thomas's Hospital, London, England*
Professor H. J. Tagnon, *Institut Jules-Bordet, Brussels, Belgium*
Dr R. M. Taylor, *National Cancer Institute of Canada, Toronto, Canada*
Dr D. Tong, *Guy's Hospital, London, England*
Dr V. Ujhazy, *Cancer Research Institute, Bratislava, Czechoslovakia*
Professor F. de Waard, *Instituut v. Sociale Geneeskunde de Rijksuniversiteit, Utrecht, Holland*
Dr T. A. Watson, *Ontario Cancer Foundation, London, Canada*
Dr B. van der Werf-Messing, *International Union Against Cancer, Geneva, Switzerland*
Dr A. Winkler, *World Health Organization, Geneva, Switzerland*
Dr P. J. Winter, *Guy's Hospital, London, England*

This document results from a meeting in Geneva on 16–18 June, 1975, supported in part by the Ontario Cancer Treatment and Research Foundation, Toronto, Canada.

### REFERENCES

BREAST CANCER GROUP IN JAPAN (1973) The Effect of Endocrine Treatment on Advanced Breast Cancer in Japan. *Jap. J. clin. Oncol.*, **6**, 13.

BRITISH BREAST GROUP (1974) Assessment of Response to Treatment in Advanced Breast Cancer. *Lancet*, ii, 38.

EASTERN CO-OPERATIVE ONCOLOGY GROUP. P. P. CARBONE, personal communication.

HAYWARD, J. L. (1966) Assessment of Response to Treatment at Guy's Hospital Breast Clinic. In *Clinical Evaluation in Breast Cancer*. Eds. J. L. Hayward and R. D. Bulbrook. London and New York: Academic Press. p. 131.

INTERNATIONAL UNION AGAINST CANCER (1974) TNM Classification of Malignant Tumours. Geneva.

KARNOFSKY, D. A. & BURCHENAL, J. H. (1948) Clinical Evaluation of Chemotherapeutic Agents in Cancer. In *Evaluation of Chemotherapeutic Agents*. Ed. C. M. MacLeod. London: Columbia University Press. p. 191.

ROZENCWEIG, M., HEUSON, J. C. (1975) Breast Cancer: Prognostic Factors and Clinical Evaluation. In *Cancer Therapy: Prognostic Factors and Criteria of Response*. Ed. M. J. Staquet. New York: Raven Press. p. 139.

SEGALOFF, A. (1966) Assessment of Response to Treatment by the Co-Operative Breast Cancer Group. In *Clinical Evaluation in Breast Cancer*. Eds. J. L. Hayward and R. D. Bulbrook. London and New York: Academic Press. p. 125.